# The influence of corneal ablation patterns on prediction error after cataract surgery in post-myopic-LASIK eyes

**DOI:** 10.1186/s40662-022-00295-1

**Published:** 2022-07-01

**Authors:** Yunqian Yao, Jing Zhao, Jifeng Yu, Wenwen He, Ling Wei, Xingtao Zhou, Yi Lu, Xiangjia Zhu

**Affiliations:** 1 Eye Institute and Department of Ophthalmology, Eye & ENT Hospital, Fudan University, 83 Fenyang Road, Shanghai, 200031 China; 2 National Health Center Key Laboratory of Myopia (Fudan University), Key Laboratory of Myopia, Chinese Academy of Medical Sciences, Shanghai, China; 3 Shanghai Key Laboratory of Visual Impairment and Restoration, Shanghai, China; 4 Shanghai Research Center of Ophthalmology and Optometry, Shanghai, China; 5 Department of Ophthalmology, Beijing Children’s Hospital, National Center for Children’s Health, Capital Medical University, Beijing, China; 6 State Key Laboratory of Medical Neurobiology, Fudan University, Shanghai, China

**Keywords:** Cataract surgery, Laser in situ keratomileusis, Intraocular lens power, Keratometry, Ablation zone decentration, Ablation zone size

## Abstract

**Purpose:**

To evaluate the influence of corneal ablation patterns on the prediction error after cataract surgery in post-myopic-LASIK eyes.

**Methods:**

Eighty-three post-myopic-LASIK eyes of 83 patients that underwent uneventful cataract surgery were retrospectively included. Predicted postoperative spherical equivalence (SE) was calculated for the implanted lens using the Haigis-L and Barrett True-K formula. Prediction error at one month postsurgery was calculated as actual SE minus predicted SE. For each eye, area and decentration of the ablation zone was measured using the tangential curvature map. The associations between prediction errors and corneal ablation patterns were investigated.

**Results:**

The mean prediction error was − 0.83 ± 1.00 D with the Haigis-L formula and − 1.00 ± 0.99 D with the Barrett True-K formula. Prediction error was positively correlated with keratometry (K) value and negatively correlated with ablation zone area using either formula, and negatively correlated with decentration of the ablation zone using the Barrett True-K formula (all *P* < 0.05). In the K < 37.08 D group, prediction error was negatively correlated with decentration of the ablation zone with both formulas (all *P* < 0.05). Multivariate analysis showed that with the Haigis-L formula, prediction error was associated with axial length (AL), K value and decentration, and with the Barrett True-K formula, prediction error was associated with AL and decentration (all *P* < 0.05).

**Conclusion:**

A flatter cornea, larger corneal ablation zone and greater decentration will lead to more myopic prediction error after cataract surgery in post-myopic-LASIK eyes.

## Background

Myopic laser refractive surgery based on the principle of corneal ablation has progressed over the past decades, and those who previously underwent myopic laser-assisted in situ keratomileusis (LASIK) have now gradually come to the age of cataract formation [1, 2]. However, it remains a great challenge for the cataract surgeons to precisely calculate the intraocular lens (IOL) power for these patients, contradicting with their high expectations of spectacle independence after cataract surgery.

Selecting an IOL power calculation formula of high accuracy is a direct and convenient way for most ophthalmologists. In recent years, new formulas, such as the Haigis-L and Barrett True-K formulas, have improved the outcomes of these challenging eyes [1, 3]. However, the percentage of patients reaching the emmetropic range (± 0.5 D) after cataract surgery hardly exceeds 60% [4]. Extreme refractive errors still occur frequently in some cases [5], which is a cause for frustrations that surgeons face.

Previous investigations found that corneal asphericity was associated with prediction errors after cataract surgery in post-LASIK eyes, indicating the potential influence of corneal ablation patterns on the predictive accuracy of IOL power calculation formulas [6, 7]. In our clinical practice, we looked carefully into patients with extreme refractive errors and noticed that the amount of ablation and ablation zone decentration may have a great influence on IOL power calculation of these eyes. However, few studies have investigated this.

Therefore, the purpose of this study was to evaluate the influence of corneal ablation patterns on prediction errors in post-myopic-LASIK eyes, and to help with predicting refractive outcomes by optimizing formulas.

## Methods

This retrospective study was affiliated with the Shanghai High Myopia Study and registered at www.clinicaltrials.gov (accession number NCT03062085). This was approved by the Institutional Review Board of the Eye and Ear, Nose, Throat (EENT) Hospital of Fudan University (No. 2013021). The study adhered to the tenets of the Declaration of Helsinki. Informed consents for the use of their clinical data were obtained from all included patients before surgery.

### Patients

Post-myopic-LASIK eyes that underwent uneventful phacoemulsification and IOL implantation during January 2019 to August 2020 at the EENT Hospital were included. Patients were excluded if they had: (1) ocular diseases that could potentially influence the postoperative refraction (e.g., keratoconus, glaucoma, zonular weakness, uveitis, severe retinopathies); (2) previous trauma or eye surgery other than corneal refractive surgery, (3) severe complications during or after cataract surgery, (4) best-corrected visual acuity (BCVA) worse than 20/40. Finally, 83 eyes of 83 patients were included in the analysis.

### Preoperative examinations

Preoperatively, all patients underwent routine ophthalmic examinations including visual acuity, slit-lamp examination, fundoscopy, B-scan ultrasonography, and biometry measurement (IOLMaster 700, Carl Zeiss Meditec AG, Jena, Germany). Corneal topography was measured with a rotating Scheimpflug camera (Pentacam HR, Oculus Optikgeräte GmbH, Wetzlar, Germany) by a single, experienced operator. Patients were asked to open both eyes and fixate at the illuminant during the measurement. Only the measurements showing “OK” in the quality specification window were qualified for analysis, otherwise the examinations would be repeated. The IOL power was calculated with the Haigis-L formula available in the IOLMaster 700 system and further validated with the Barrett True-K formula (no history) formula available on the ASCRS website.

### Surgical procedures

A single experienced surgeon (YL) performed all the surgeries using a standard procedure. The cataract was removed by phacoemulsification through a 2.6 mm clear corneal microincision, followed by the implantation of a negative aberration aspheric IOL (MC X11 ASP, HumanOptics, Germany) in the capsular bag. Residual viscoelastics (DisCoVisc; Alcon Laboratories, USA) were then removed from above and below the IOL, and the incision was hydrated followed by cephalosporin injection into the anterior chamber through lateral corneal incisions. Postoperatively, all patients received the same anti-inflammatory treatments.

### Postoperative examinations

Uncorrected visual acuity [UCVA, logarithm of the minimal angle of resolution (logMAR)], BCVA (logMAR) and manifest refraction were assessed at one month after cataract surgery. For each eye, the predicted postoperative spherical equivalence (SE) was calculated for the implanted lens using both the Haigis-L and Barrett True-K formulas. The lens constants for the IOLMaster 700 reported on the User Group for Laser Interference Biometry (ULIB) website were used. The prediction error was then calculated as the actual postoperative SE minus the predicted SE for both formulas. The mean absolute errors (MAEs) and the median absolute errors (MedAEs) were subsequently calculated for each formula.

### Evaluation of corneal ablation patterns

**Fig. 1:**
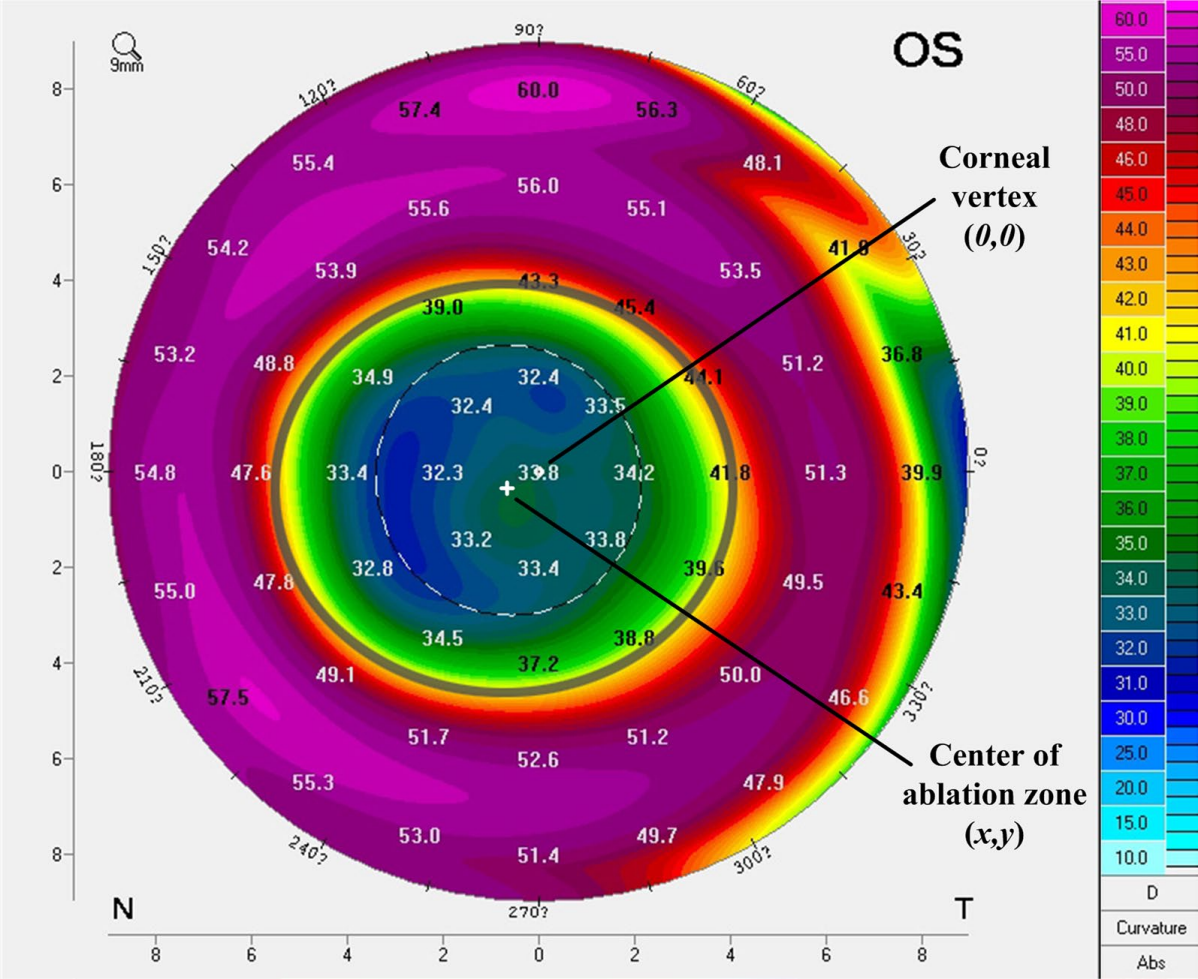
Schematic diagram showing the measurement of ablation zone decentration using the tangential curvature (front) map acquired with the Pentacam HR. The edge of the ablation zone is manually outlined as indicated by decreased tangential curvature (the color change from yellow to red). The area within the fitted circle or ellipse is the ablation zone. Decentration is measured as the distance from the fitted ablation center (x, y) (white cross) to corneal vertex (0,0) (white dot) as referred to the scale at the bottom or on the left side of the map

K values measured with the IOLMaster 700 before cataract surgery were recorded. For each eye, the decentration of ablation zone was measured manually using the tangential curvature map acquired with the Pentacam HR. As is shown in Fig. 1, the area within the fitting circle or ellipse on the anterior surface indicated by decreased tangential curvature was considered the ablation zone [8], and its center (x, y) was regarded as the center of corneal ablation [9]. Ablation zone diameters and areas were measured under the same color scale settings. Decentration was calculated as the distance ($\sqrt{{x}^{2}+{y}^{2}}$) between the fitting ablation center and the corneal vertex (0, 0). Two experienced (YY and JZ) independently performed all measurements. For each eye, the agreement of measurements was assessed, and the average value of ablation zone area and decentration was used for statistical analysis. Any inconsistency was discussed and resolved under the guidance of a third doctor (XZ).

### Statistical analysis

Statistical analysis was performed using SPSS Statistics 23 (IBM, Chicago, USA). Continuous variables were described as mean ± standard deviation (SD). Paired t*-*tests were used to compare the visual acuities before and after cataract surgery. Percentage of eyes within certain range of prediction error using two formulas was compared with Chi-square test. The absolute errors of two formulas were compared using the related-samples Wilcoxon signed-rank test. Bland-Altman plots were used to examine the agreement of measured area and decentration between doctors. Pearson correlation analysis was performed for assessing the relationships between corneal ablation patterns and prediction errors, while Spearman correlation analysis was used for evaluating relationships between corneal ablation patterns and absolute errors. The backward stepwise multiple linear regression analysis was further conducted to identify the influential factors on prediction errors in post-myopic-LASIK eyes. For all analyses, two-sided *P* values less than 0.05 were considered statistically significant.

## Results

### Patient characteristics

Demographic data of the patients are presented in Table 1. UCVA and BCVA improved significantly at one month after cataract surgery (paired t-test, all* P* < 0.001).

**Table 1:** Patient demographics and characteristics

Characteristic	Ratio/mean ± SD (range)
Age (years)	53.3 ± 8.6 (35.0–76.0)
Sex (male/female)	38/45
Eye laterality (left/right)	40/43
Axial length (mm)	29.84 ± 2.05 (25.57–33.91)
K value (D)	36.86 ± 1.91 (32.49–41.28)
Preoperative visual acuity	
UCVA (logMAR)	0.98 ± 0.63 (3.00–0.22)
BCVA (logMAR)	0.79 ± 0.38 (1.70–0.22)
Postoperative visual acuity	
UCVA (logMAR)	0.59 ± 0.40 (1.30–0.00)
BCVA (logMAR)	0.18 ± 0.10 (0.30–0.00)

*SD =* standard deviation; *UCVA* = uncorrected visual acuity; *BCVA* = best-corrected visual acuity; *D* = diopter; *logMAR* = logarithm of the minimum angle of resolution

### Prediction errors after cataract surgery

**Fig. 2:**
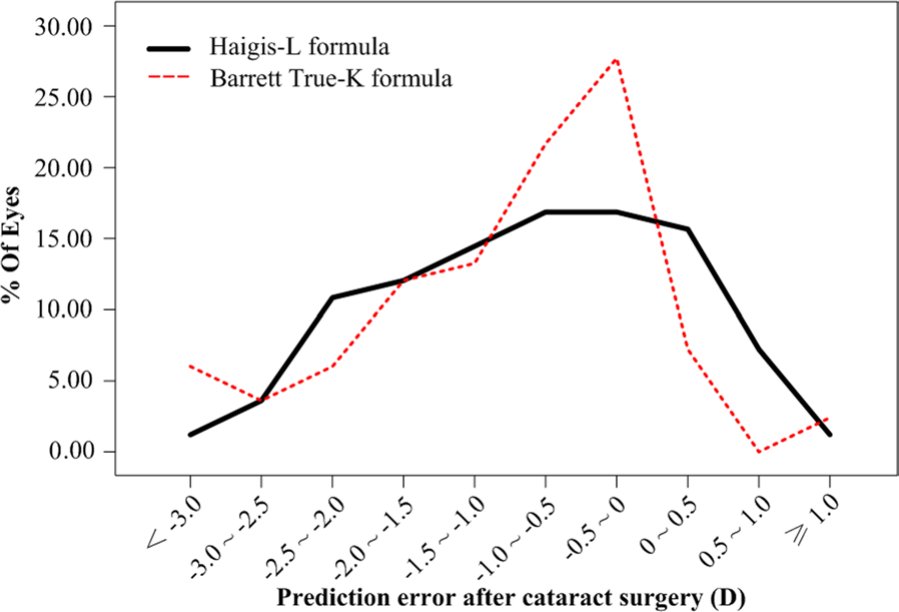
Distribution of postoperative refractive errors with two formulas. With the Haigis-L formula, 32.5% (27/83) and 56.6% (47/83) of eyes had a prediction error within ± 0.50 D and ± 1.00 D, respectively. With the Barrett True-K formula, the percentages were 34.9% (29/83) and 57.8% (48/83), respectively. More eyes had a prediction error between − 1.00 to 0.00 D with the Barrett True-K formula than the Haigis-L formula (41/83 *vs.* 28/83, Chi-squared test, *P* = 0.041). D, diopter

The mean prediction error was − 0.83 ± 1.00 D (range, − 3.14 to 1.27 D) with the Haigis-L formula, and − 1.00 ± 0.99 D (range, − 3.67 to 1.09 D) with the Barrett True-K formula. With the Haigis-L formula, 32.5% (27/83) and 56.6% (47/83) of eyes had a prediction error within ± 0.50 D and ± 1.00 D, respectively. With the Barrett True-K formula, the percentages were 34.9% (29/83) and 57.8% (48/83), respectively (Fig. 2). More eyes had a prediction error between − 1.00 to 0.00 D with the Barrett True-K formula than the Haigis-L formula (41/83 *vs.* 28/83, Chi-square test, *P* = 0.041).

The MAE was 1.04 ± 0.78 D with the Haigis-L formula and 1.07 ± 0.91 D with the Barrett True-K formula. The MedAE was 0.91 D with the Haigis-L formula and 0.86 D with the Barrett True-K formula. No significant difference was identified in MedAE between the two formulas (related-samples Wilcoxon signed-rank test, *P* = 0.823).

### Corneal ablation patterns and prediction errors

**Fig. 3:**
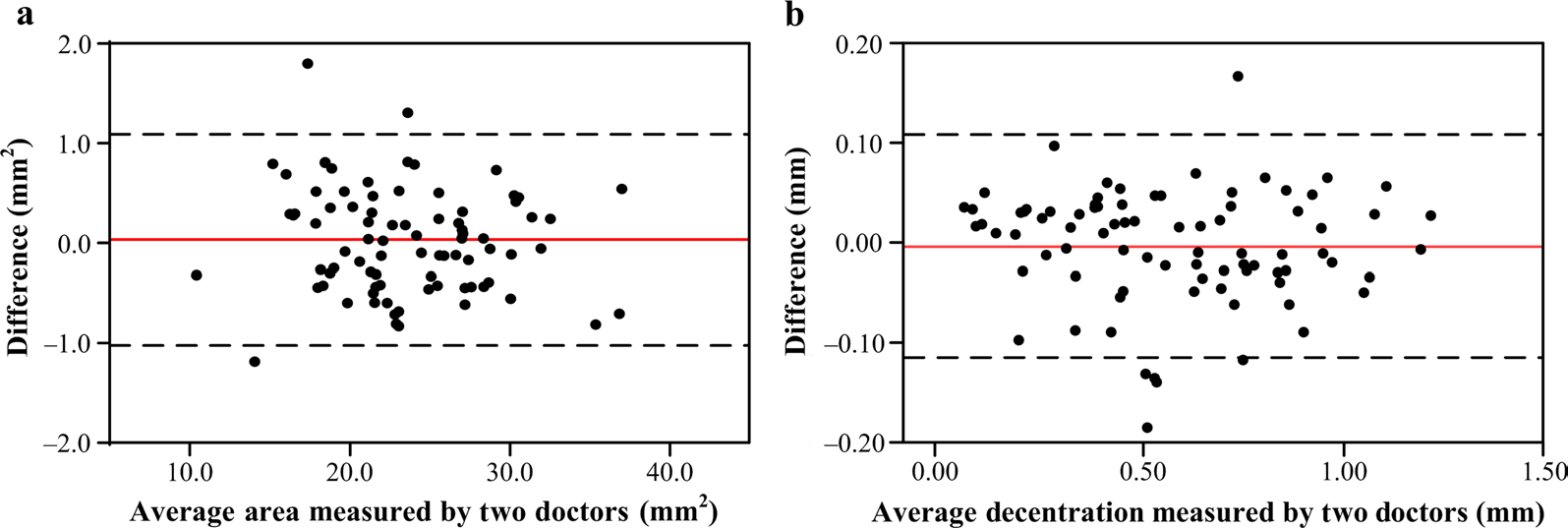
Bland-Altman plots for ablation zone area and decentration measured by two doctors independently. Dotted lines denote limits of agreement (LOA). The horizontal line in the middle denotes the mean of difference between measurements. **a** For ablation zone area, 80 (96.4%) of 83 eyes were within LOA (range, − 1.022 to 1.090 mm^2^), and the mean of difference was 0.034 mm^2^. **b** For decentration, 77 (92.8%) of 83 eyes were within the LOA (range, − 0.115 to 0.109 mm), and the mean of difference was − 0.003 mm

The mean long and short axes diameter of the ablation zone was 5.68 ± 0.65 mm (range, 4.19 to 7.21 mm) and 5.33 ± 0.67 mm (range, 3.37 to 6.99 mm), respectively. The mean area of the ablation zone was 24.18 ± 5.36 mm^2^ (range, 10.95 to 38.10 mm^2^). The mean decentration of the ablation zone was 0.59 ± 0.29 mm (range, 0.08 to 1.24 mm). Figure 3 shows the reproducibility of ablation zone area and decentration measurement.

**Fig. 4:**
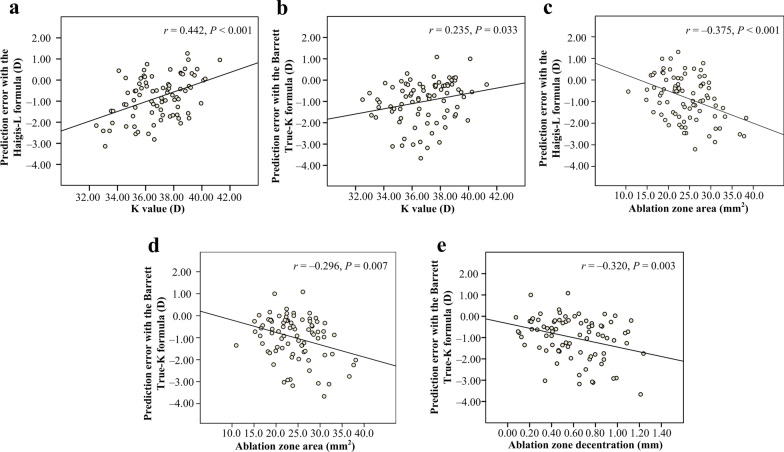
Correlations between prediction error and corneal ablation patterns in post-myopic-LASIK eyes. Prediction error was positively related to K value (**a** Haigis-L formula, Pearson’s* r* = 0.442, *P* < 0.001; **b** Barrett True-K formula, Pearson’s* r* = 0.235, *P* = 0.033) and negatively related to ablation zone area (**c** Pearson’s *r* = − 0.375, *P* < 0.001 for Haigis-L formula; **d** Pearson’s *r* = − 0.296, *P* = 0.007 for Barrett True-K formula) with both formulas. With the Barrett True-K formula, prediction error was negatively correlated with ablation zone decentration (**e** Pearson’s *r* = − 0.320, *P* = 0.003). D, diopter

Prediction error was positively correlated with K value (Pearson’s *r* = 0.442, *P* < 0.001 for Haigis-L formula, and Pearson’s *r* = 0.235, *P* = 0.033 for Barrett True-K formula) (Fig. 4a, b) and negatively correlated with ablation zone area (Pearson’s *r* =  − 0.375, *P* < 0.001 for Haigis-L formula, and Pearson’s *r* =  − 0.296, *P* = 0.007 for Barrett True-K formula) (Fig. 4c, d) with both formulas. Furthermore, prediction error was negatively correlated with decentration with Barrett True-K formula (Pearson’s *r* = − 0.320, *P* = 0.003) (Fig. 4e).

**Fig. 5:**
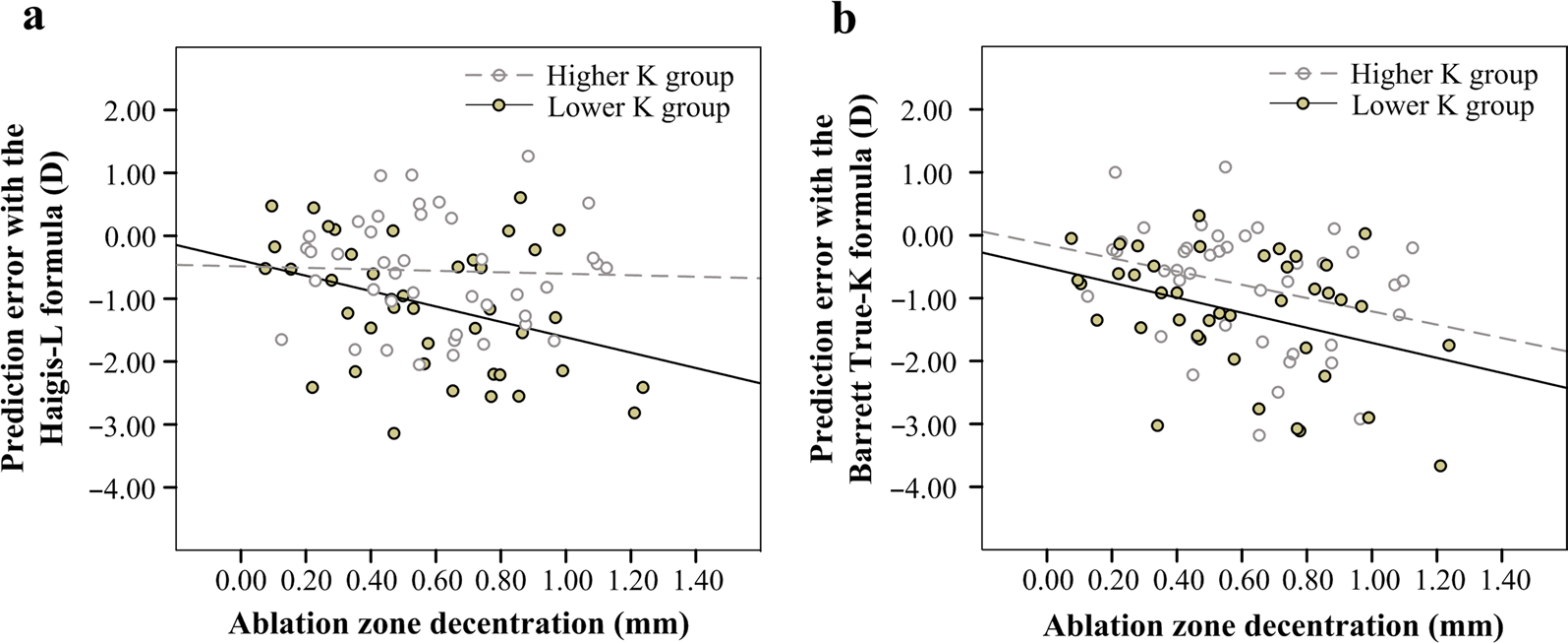
Correlations between prediction error and ablation zone decentration in post-myopic-LASIK eyes. In the lower K group (K value < 37.08 D), prediction error was negatively correlated with decentration with both formulas (**a** Haigis-L formula, Pearson’s *r* = − 0.353, *P* = 0.023; **b** Barrett True-K formula, Pearson’s* r* = − 0.369, *P* = 0.018). However, in the higher K group (K value ≥ 37.08 D), prediction error was not significantly correlated with decentration no matter which formula was used (all *P* > 0.05)

To better understand the effect of corneal ablation patterns on prediction errors, Pearson correlation analysis was further performed after stratification by medium K value (37.08 D): in the lower K group (K value < 37.08 D), prediction error was negatively correlated with decentration with both formulas (Haigis-L formula, Pearson’s *r* = − 0.353, *P* = 0.023; Barrett True-K formula, Pearson’s *r* =  − 0.369, *P* = 0.018, respectively). However, in the higher K group (K value ≥ 37.08 D), prediction error was not significantly correlated with decentration no matter which formula was used (all *P* > 0.05) (Fig. 5).

The backward stepwise multiple linear regression analysis, which included age, gender, eye laterality, axial length (AL), K value, ablation zone area and decentration demonstrated that, with the Haigis-L formula, prediction error was associated with AL (*β* = 0.273, *P* = 0.027), K value (*β* = 0.601, *P* < 0.001) and decentration (*β* =  − 0.327, *P* = 0.002). With the Barrett True-K formula, prediction error was only associated with AL (*β* = − 0.326, *P* = 0.003) and decentration (*β* = − 0.212, *P* = 0.049).

### Corneal ablation patterns and absolute errors

Spearman correlation analysis was further performed to evaluate the influence of corneal ablation patterns on absolute errors. Absolute error was negatively correlated with K value (Spearman’s *r* =  − 0.290, *P* = 0.008 for Haigis-L formula, and Spearman’s *r* =  − 0.239, *P* = 0.030 for Barrett True-K formula), and positively correlated with ablation zone area (Spearman’s *r* = 0.313, *P* = 0.004 for Haigis-L formula, and Spearman’s *r* = 0.253, *P* = 0.021 for Barrett True-K formula) and decentration (Spearman’s *r* = 0.284, *P* = 0.009 for Haigis-L formula, and Spearman’s *r* = 0.260, *P* = 0.018 for Barrett True-K formula).

## Discussion

Accurate IOL power calculation is of great importance and difficulty for eyes that have undergone corneal refractive surgery, attracting the attention of cataract surgeons all over the world. Three factors are known to contribute to inaccurate predictions: keratometric index error (incorrect keratometric index induced by the change in relationship between the anterior and posterior cornea), radius error (inaccurate curvature measurements), and formula error (incorrectly estimated lens position) [1, 4]. To overcome these problems, more than 30 optimized methods have been proposed in the past decades [5], among which, the Haigis-L and Barrett True-K formulas achieved good results as reported in previous studies [4]. However, extreme refractive errors still occur occasionally. Although the accuracy of these formulas has been widely compared and analyzed, there was a lack of investigations into the potential contributing factors on refractive surprises in their practical applications. Here, we evaluated the influence of corneal ablation patterns on IOL power calculation in post-myopic-LASIK eyes, and found that a flatter cornea, larger ablation zone and greater decentration of prior corneal laser ablation were associated with increased myopic refractive error after cataract surgery. However, the influence of decentration decreases with fewer ablations.

In our study, neither of the two formulas were found to have ideal prediction accuracy, and only less than 40% of eyes were within ± 0.5 D of predicted refraction with both formulas. This rate was consistent with the 28.21% to 68% for no history methods reported by previous studies [2, 10–13], but significantly lower than the 69.6% to 80.8% in virgin eyes [14]. While underestimation of IOL power and consequent hyperopic outcomes after cataract surgery often occur in post-myopic-LASIK eyes when using normal formulas [15], myopic prediction errors were frequently reported with some modified formulas [3, 11, 13]. In this study, the proportion of eyes with prediction error greater than − 1.0 D was high and almost coincident between the two formulas, indicating their common defect in avoiding great refractive surprises thereby not being able to detected some underlying influential factors. Furthermore, we found that the distribution of prediction errors was different between the two formulas, suggesting that the accuracy of the two formulas might be influenced by different factors.

Notably, our study identified a more myopic prediction error correlated with flatter corneas and larger ablation zones in post-myopic-LASIK eyes using both formulas. While a lower K value and a larger ablation zone both reflect a larger amount of myopic correction [16–18], it indicates a potential effect of ablation amount on formula accuracy. In tandem, Vrijman et al. suggested that when the excimer laser correction of myopia exceeded 6.0 D, more myopic prediction errors and higher absolute errors were obtained after cataract surgery [19]. There are several reasons which may explain the result: (1) The ASCRS formulas developed linear regression models based on a small sample size (e.g., 40 eyes of 20 patients for the Haigis-L formula [1]) and a limited range of myopic correction or corneal power, and thus those eyes with extreme K values may not be in accordance with the calculated linear regression relationship [4, 20]; (2) The ratio ΔK/ΔSE decreases to less than one with the increase in the amount of myopic correction [20, 21], therefore, effective K value (equaling the preoperative K value minus ΔSE at the corneal plane [22, 23]) derived from the refractive history method might be smaller than the actual K value for more-ablated corneas, leading to an overestimation of IOL power and a myopic prediction error after cataract surgery. These may explain why K value significantly influenced the accuracy of the Haigis-L, which is known to develop regression models based on the refractive history method [1, 4], and the Barrett True-K formulas in our study population.

Other factors may also account for the size of the ablation zone. As the central corneal curvature is usually determined by paracentral measurements, for small ablation zones, it is more likely to be measured on the periphery of treated zones [5], and thus lead to the steepness of corneal curvature gradient across the ablation zones and for an overestimated central corneal curvature, resulting in more hyperopic outcomes as confirmed in our study. However, while the ablation zone area and K value both reflect the amount of myopic correction, K value exhibited more robust predictability for IOL power calculation according to the multivariable regression analysis.

**Fig. 6:**
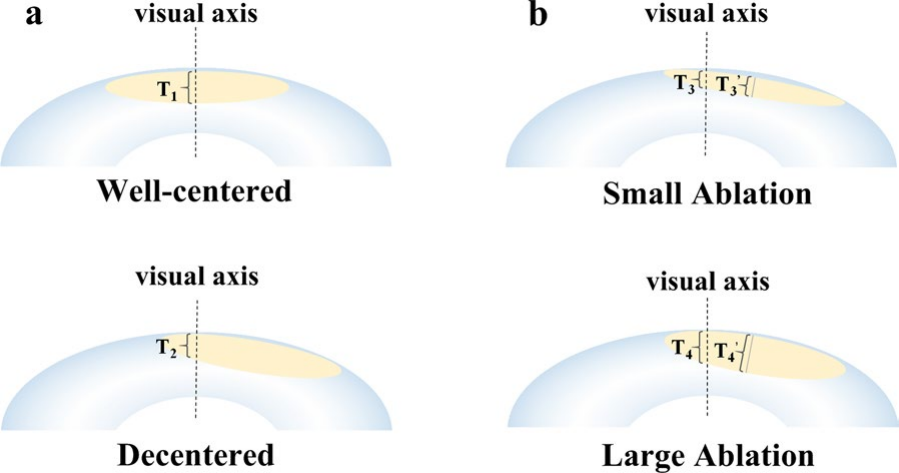
Schematic diagrams showing the influence of corneal ablation patterns on prediction errors after cataract surgery in post-myopic-LASIK eyes. T_1_, T_2_, T_3_ and T_4_ refer to the thickness of the ablation zone at the corneal vertex, while T_3_^’^ and T_4_^’^ refer to the ablation center. **a** The left two diagrams show a well-centered and a decentered ablation with equal ablation amount. When the ablation is decentered, the thickness of the ablation zone at the corneal vertex is thinner than expected (T_2_ < T_1_). Therefore, the relationship between the anterior and posterior corneal surfaces has further changed, leading to extra errors of available formulas which are based on well-centered ablation models. **b** The right two diagrams show different number of ablations with equivalent magnitude of decentration. When the amount of ablation is small, the variation in the thickness across the ablation zone is limited (δT_3_ < δT_4_; δT_3_ = T_3_^’^ − T_3_, δT_4_ = T_4_^’^ − T_4_). Therefore, the change in the relationship between anterior and posterior corneal surfaces induced by ablation zone decentration and its consequent influence on prediction errors might be negligible. However, when the amount of ablation is large, its effects should not be ignored

One of the most interesting findings of this study is that ablation zone decentration had a significant influence on IOL power calculation, which has long been overlooked in previous investigations. Decentered ablation is among the most common complications of corneal refractive surgery, resulting from the misalignment of eye-tracking systems, large angle kappa, pupil center shifts after dilation, etc. [24]. In this study, the tangential curvature map was used for the evaluation of ablation zone decentration. It can correctly highlight the edge of the ablation zone, the site of highest curvature change, which has been recognized as a reliable way to evaluate the ablation profile when only post-LASIK topography is available [9, 25]. Several reasons may explain this: (1) Extra keratometric index errors. When the ablation is decentered, the thickness of the ablation zone at the corneal vertex is thinner than expected. Therefore, the relationship between the anterior and posterior corneal surfaces has further changed [26–28], leading to extra errors of available formulas which are based on well-centered ablation models (Fig. 6a); (2) Extra radius errors. The IOLMaster measures the corneal curvature by analyzing six symmetrical light reflections projected onto the around 2.5 mm ring of the anterior corneal surface [29], therefore, decentered ablation will result in an inaccurate measurement of anterior corneal curvature because of the large variations of corneal curvatures within the irregular central cornea; (3) Greater ablation zone decentration will lead to a higher irregular corneal astigmatism which can be difficult to correct by cataract surgery [24, 30].

Of note, such an association exists in eyes with flatter corneas, but disappears with steeper corneas, indicating that the influence of decentration might depend on a particular level of the amount of ablation. This is consistent with our clinical observations that IOL power can still be accurately estimated in some prominently decentered cases, where a commonality exists in their topography—small amount of ablation as indicated by few concentric rings on the elevation map coupled with steeper central corneal surface. This can be due to the limited variation in the thickness of the ablation zone when the amount of myopic correction is small. Therefore, the change in the relationship between the anterior and posterior corneal surfaces induced by ablation zone decentration and its consequent influence on prediction errors could be negligible (Fig. 6b). However, when the amount of ablation is large, its effects could not be ignored.

## Conclusions

In conclusion, our study demonstrated that with a corneal topographic system, the patterns of prior laser ablation could be evaluated, and flatter corneas, larger ablation zone and greater decentration might contribute to more myopic prediction errors after cataract surgery in post-myopic-LASIK eyes. This should allow the individual refractive outcome to be predictable, providing information for preoperative communication and formula optimization.

## Data Availability

The data that support the findings of this study are available from the corresponding author upon reasonable request.
